# Concordance of Alzheimer’s Disease Subtypes Produced from Different Representative Morphological Measures: A Comparative Study

**DOI:** 10.3390/brainsci12020187

**Published:** 2022-01-30

**Authors:** Baiwen Zhang, Lan Lin, Lingyu Liu, Xiaoqi Shen, Shuicai Wu

**Affiliations:** 1 Department of Biomedical Engineering, Faculty of Environment and Life Sciences, Beijing University of Technology, Beijing 100124, China; zhangby@emails.bjut.edu.cn (B.Z.); llyy123llyy@sina.com (L.L.); xiaoqishen@emails.bjut.edu.cn (X.S.); 2 Intelligent Physiological Measurement and Clinical Translation, Beijing International Base for Scientific and Technological Cooperation, Beijing University of Technology, Beijing 100124, China

**Keywords:** Alzheimer’s disease, gray matter density, cortical thickness, structural magnetic resonance imaging, matched Alzheimer’s disease subtypes

## Abstract

Background: Gray matter (GM) density and cortical thickness (CT) obtained from structural magnetic resonance imaging are representative GM morphological measures that have been commonly used in Alzheimer’s disease (AD) subtype research. However, how the two measures affect the definition of AD subtypes remains unclear. Methods: A total of 180 AD patients from the ADNI database were used to identify AD subgroups. The subtypes were identified via a data-driven strategy based on the density features and CT features, respectively. Then, the similarity between the two features in AD subtype definition was analyzed. Results: Four distinct subtypes were discovered by both density and CT features: diffuse atrophy AD, minimal atrophy AD (MAD), left temporal dominant atrophy AD (LTAD), and occipital sparing AD. The matched subtypes exhibited relatively high similarity in atrophy patterns and neuropsychological and neuropathological characteristics. They differed only in MAD and LTAD regarding the carrying of apolipoprotein E ε2. Conclusions: The results verified that different representative morphological GM measurement methods could produce similar AD subtypes. Meanwhile, the influences of apolipoprotein E genotype, asymmetric disease progression, and their interactions should be considered and included in the AD subtype definition. This study provides a valuable reference for selecting features in future studies of AD subtypes.

## 1. Introduction

It is generally accepted that the neurofibrillary tangles (NFTs) of Alzheimer’s disease (AD) derive from the entorhinal cortex, then spread subsequently to the association cortex via the hippocampus, and finally invade the primary cortex [1]. However, several recent studies have suggested that AD patients have striking differences in neuropathologic distinctions, cognitive functions, demographics, and clinical progression, indicating that AD is a heterogeneous disease with different subtypes [2,3]. The definition of the AD subtype is an essential part of capturing the heterogeneity. As proved by some studies, the gray matter (GM) change in brain region is closely associated with tau pathology in AD patients with abnormal amyloids [4]. Therefore, GM atrophy pattern-based morphometric measures have received increasing attention in the studies of biologically defined AD subtypes [5,6].

Given the high-dimensionality of structural magnetic resonance imaging (sMRI), automated region-based analyses have been conducted in the studies of AD subtypes by parcellating the brain into anatomically defined regions. GM density and cortical thickness (CT) obtained from sMRI are two widely used morphometric features. Both GM features respectively constitute multimode features with pathological information, so they have been applied to studies on AD subtypes [7,8]. The GM density for each region-of-interest (ROI) is computed as the sum of GM densities of all voxels within the ROI from the voxel-based morphometry (VBM) maps using statistical parametric mapping (SPM). The surface-based CT value is obtained by averaging the vertex CT values within all FreeSurfer cortical ROIs [9,10]. The study results may be influenced by the technical differences in VBM and FreeSurfer, both of which rely on ROIs parcellation that uses a predefined template to identify morphometric changes, but defined ROIs are quite different. CT is one of the hallmark features of AD, and a greater degree of cortical thinning in a disease-affected region indicates the higher severity of AD [11]. However, CT analysis fails to detect the abnormalities in subcortical areas such as the hippocampus [12]. Furthermore, in the early stage of cognitive decline, CT analysis may be relatively insensitive to the abnormalities of some medial temporal lobe (MTL) regions [13]. The density-based approach provides complementary information to the surface-based analysis, thus effectively probing the atrophied subcortical structures [14]. In several density-based studies, the MTL AD subtype experiences severe atrophy in parts of the limbic system such as the hippocampus and other subcortical ROIs [15,16,17], which is also verified by the higher distribution of tau in the hippocampus in Murray’s study [2].

These two morphological measures may be correlated to some extent, where CT measures cortical thinning, and the change in GM density measures a mixed change in local CT, cortical folding, and gyrification. It has been suggested in some studies that CT can drive facilitate the change in GM density [18]. However, most previous studies on AD subtypes definition have focused on only one morphological feature. In our earlier study, four AD subtypes were successfully defined based on the CT features [19], among which each subtype presented its own neuropsychological and neuropathological characteristics. Although several distinct AD subtypes have been identified in most studies, they differ in the definition of AD subtypes, which may be partly attributed to different representative morphological measures [3,19]. This raises a crucial question: How do different GM measurement methods affect the definition of AD subtypes? To answer this question, in the present study, AD subtypes were defined by density-based and CT-based features, respectively, for AD patients from the Alzheimer’s disease Neuroimaging Initiative (ADNI) database. If the matched subtypes could be generated by different measurement methods, the characteristics of matched subtypes will be further explored. To our knowledge, this study takes the lead in comparing the two GM morphological measures in AD subtype definition.

## 2. Materials and Methods

### 2.1. Subjects and MRI Processing

The ADNI project was launched in 2004 [20]. The first phase (ADNI-1) lasted from 2004 to 2010, during which time the plan was to recruit a total of 800 individuals aged 50–90, including AD: 200, cognitive normal (CN): 200, and mild cognitive impairment (MCI): 400. The information of all participants of this project was derived from ADNI-1 baseline scans (adni.loni.usc.edu). The T1-weighted images were acquired with a 1.5 T MRI scanner. The scanning parameters were as follows: TR = 3000 ms, TE = 3.55 ms, slice thickness = 1.2 mm, voxel size = 1.2 × 0.94 × 0.94 mm^3^ [21].

The density features used were acquired from ‘uaspmvbm.csv’, downloaded from the ADNI website (https://ida.loni.usc.edu/, accessed on 3 January 2022), which were extracted from VBM analysis via SPM software (version 5). A total of 116 anatomical ROIs (90 in cerebrum and 26 in cerebellum) were defined using the automated anatomical atlas (AAL) [14]. The CT features extracted by FreeSurfer version 4.3 (http://freesurfer.net/, accessed on 3 January 2022) were acquired from ‘ucsffx.csv’, downloaded from the ADNI website (https://ida.loni.usc.edu/, accessed on 3 January 2022). The thickness values are based on the Desikan-Killiany atlas, including 34 parcels per hemisphere [10]. 

**Figure 1:**
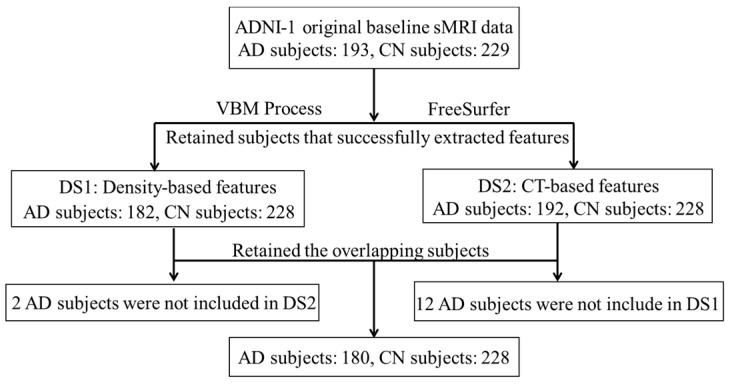
Data screening process. *Abbreviations:* DS: Data set, ADNI: Alzheimer’s disease Neuroimaging Initiative, CT: Cortical thickness, VBM: voxel-based morphometry, AD: Alzheimer’s disease, CN: cognitive normal subjects, sMRI: structural MRI.

Only the subjects having both CT and density features were kept in the study. Fourteen subjects were excluded due to inconsistencies. The screening process of subjects is shown in Figure 1.

### 2.2. Definition of AD Subtypes

**Figure 2:**
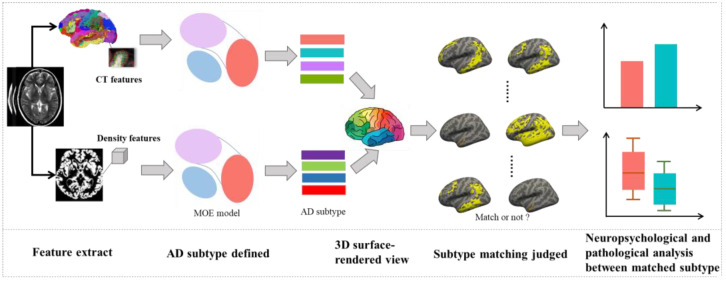
Overview of the steps for AD subtype definition and analysis. *Abbreviations:* AD: Alzheimer’ disease, MOE: mixture of experts model, CT: cortical thickness.

The features extracted from the screened subjects were input into the mixture of experts (MOE) model to define subtypes [22]. The process of subtypes definition and subsequent analysis is shown in Figure 2. 

Before the features were input into the MOE model, the age, sex, years of education, and intracranial volume (ICV) were entered as nuisance variables, so they were regressed from the original features. First, the regression coefficient of CN ($\beta_{CN}$) was calculated using a generalized linear model as Formula (1):

(1) $$density_{value_{CN}}(or\;CT_{value_{CN}})=\beta_{CN}\times (1+age_{CN}+sex_{CN}+edu_{CN}+ICV_{CN})+\varepsilon$$

Then, the effects of each nuisance variable were regressed out of all subjects, as Formula (2):

(2) $$Residual_{All}=density\_value_{all}(or\;CT\_value_{all})-\beta_{all}\times (1+age_{all}+sex_{all}+edu_{all}+ICV_{all})$$

After the regression analysis, each subject was given a binary label, $y_{i}\in \{-1,1\}$. The CN subjects were set as the reference group (*y* = −1) and the AD patients as the affected group (*y* = 1). Ten-fold cross-validation was adopted for the MOE. The MOE combined the fuzzy c-means (FCM) and support vector machines (SVM). The joint optimization model formula was as follows (3):

(3) $$minimize_{{}_{{\left\{w^{k}\right\}}_{k}{\left\{m_{i}^{k}\right\}}_{i,k}}}\sum_{k=1}^{K}{\left\{\frac{1}{2}||w^{k}{||}_{1}+C{\sum_{i=1}^{N}m_{i}^{k}(1-y_{i}{\left(w^{k}\right)}^{T}x}_{i}\right)}_{+}^{2}+t\sum_{i=1}^{N}{\left(m_{i}^{k}\right)}^{2}||x_{i}-d^{k}{||}_{F}^{2}\}$$

$$subject\;to:\sum_{k=1}^{K}m_{i}^{k}=1,m_{i}^{k}\in [0,1]\;n=1,\cdots ,N,d^{k}=\frac{\sum_{i=1}^{N}{\left(m_{i}^{k}\right)}^{\alpha }x^{i}}{\sum_{i=1}^{N}{\left(m_{i}^{k}\right)}^{\alpha }}$$

where *K* is the number of experts, *m* is the value of membership, *N* is the number of AD subjects, *C* is the loss penalty, and t is the trade-off between the cost of SVMs and FCM.

The number of experts was fixed at four. The parameters *C* and *t* were simultaneously optimized through the grid search, with a search range of {2^−3^~2^10^}, respectively. The selection of parameters mainly depended on the cross-validated accuracy (ACC), maximum pair-wise inner-product (W_r_), and Bezdek partition coefficient (BPC) [23]. Given all those assessments, the density-based AD subtypes and CT-based AD subtypes were defined, respectively. Each subtype was then named according to its atrophic regions. 

### 2.3. Statistical Analysis

The characteristics included three main parts: (1) demographic, including age, gender, years of education, onset age, and duration of AD. (2) Cognitive characteristics, including the Mini-Mental State Exam (MMSE), Clinical Dementia Rating Scale-Sum of Boxes (CDRSB), AD Assessment Scale-Cognitive Subscale (ADAS-Cog), and for subdomains ADNI composite scores: memory (ADNI-MEM), executive (ADNI-EF), language (ADNI-LAN), and visuospatial abilities (ADNI-VS) [24,25]. (3) Apolipoprotein E (APOE) genotypes and cerebrospinal fluid (CSF) biomarker abnormality levels, including APOE ε2, APOE ε4, beta-amyloid 1-42 (Aβ_1-42_), Phosphorylated tau (P-tau), and Total tau (T-tau). The cut-off value of Aβ_1-42_, T-tau, and P-tau were 192 ng/L, 93 ng/L, and 23 ng/L, respectively [26,27]. The differences between matched subtype were compared. The differences of qualitative variables were calculated via the chi-square tests. The quantitative variables were assessed using analysis of variance (ANOVA), and pairwise comparison by the Dunnett’s test. Statistical analysis was performed with SPSS, version 19.0, Armonk, NY, USA.

## 3. Results

### 3.1. Subtypes Definition and Matching

All AD subjects were divided into four subtypes. Considering all the evaluation criteria of MOE, the parameter values were reasonably selected. The optimized parameters and evaluation indicators are shown in Table 1. 

**Figure 3:**
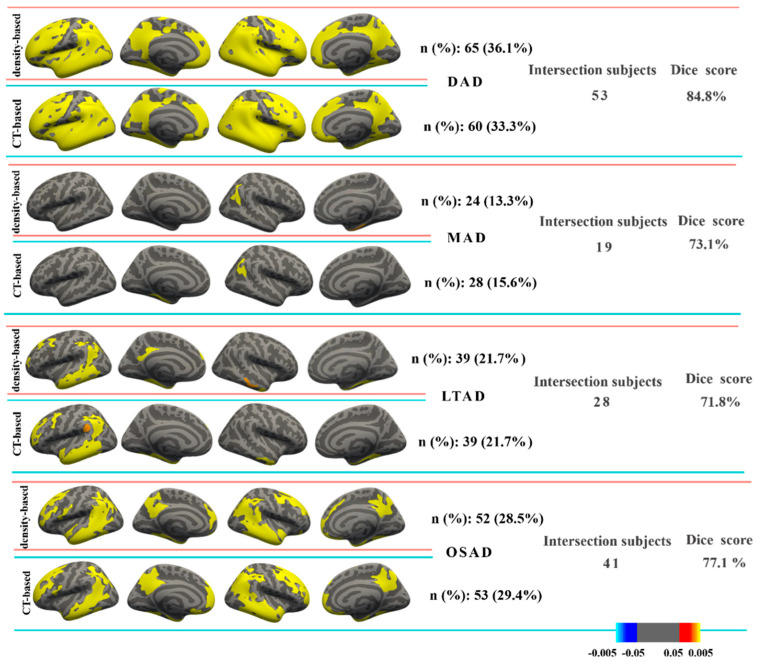
CT maps of subtypes defined by CT or density when compared with CNs. The brain maps were uncorrected for multiple comparisons at *p* < 0.05. The statistical parametric maps included all the subjects in each subtype. *Abbreviations:* DAD: diffuse atrophy AD subtype, MAD: minimal atrophy AD subtype, LTAD: left temporal dominant atrophy AD subtype, OSAD: occipital sparing AD subtype, CT: cortical thickness, n: the number of AD subjects, %: proportion of AD subjects in a subtype.

The CT difference between AD subtype and CN were rendered in the same FreeSurfer atlas to compare the differences of the match subtype (Figure 3). As can be seen from Figure 3, the four subtypes defined by two features were one-to-one matched in cortical atrophy regions, and named diffuse atrophy AD (DAD), minimal atrophy AD (MAD), left temporal dominant atrophy AD (LTAD), and occipital sparing AD (OSAD), respectively.

The subjects in the matched subtype overlapped greatly, and their Dice scores, calculated according to formula (4), ranged from 71.8% to 84.8%. The ‘Subjects’ in the formula were from the matched subtype. The number of overlapping subjects (intersection subjects) and the results of the Dice score in each matched subtype is indicated in Figure 3. The characteristics of each subtype are summarized in Table 2.

(4) $$Dice\;score=\frac{2\times |Subjects_{density-based}\cap Subject_{CT-based}|}{\left|Subjects_{density-based}|+|Subject_{CT-based}\right|}$$

### 3.2. GM Density Map between Matched Subtypes

**Figure 4:**
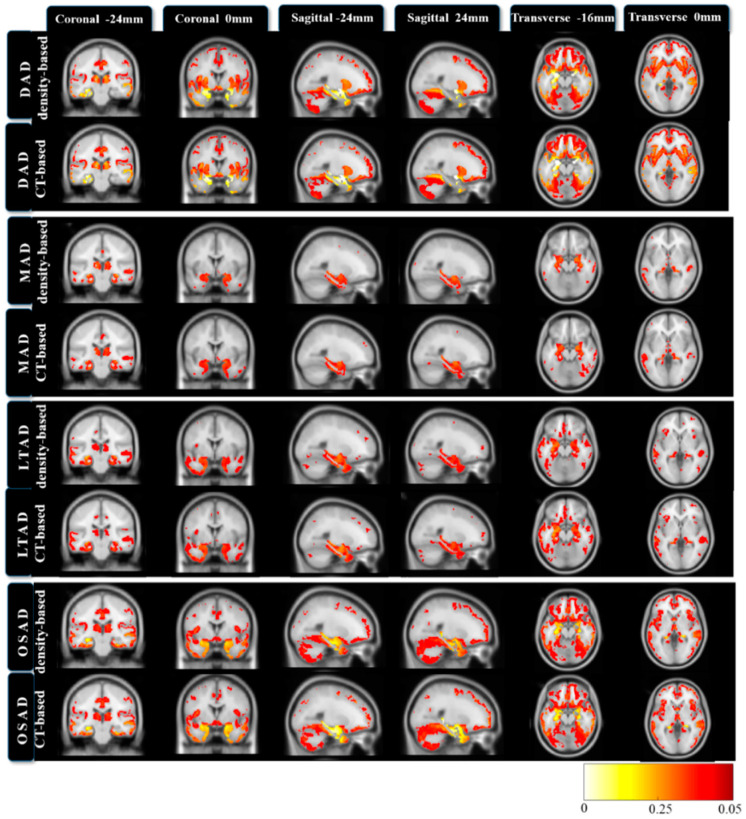
Statistical parametric maps of subtypes were identified by CT-based and density-based compared with the CNs. The results were thresholded at FDR corrected *p* < 0.05. The statistical parametric maps included all the subjects in each subtype. Abbreviations: DAD: diffuse atrophy AD subtype, MAD: minimal atrophy AD subtype, LTAD: left temporal dominant atrophy AD subtype, OSAD: occipital sparing AD subtype, CT: cortical thickness.

Figure 4 presents the statistical parametric maps between AD subtype and CN of matched subtypes. A false discovery rate (FDR)-corrected p-value threshold of 0.05 was used. The regional GM atrophy between matched subtypes were roughly consistent. The DAD exhibited extensive atrophy. In LTAD, the GM atrophy was found in the temporal lobe ROIs, such as the hippocampus, amygdala, and fusiform. The more significant difference was manifested in the left lateralized regions. The OSAD displayed the atrophy ROIs in temporal, frontal, parietal, and posterior fossa structures. Compared to the DAD, the differences between OSAD and NC were slightly smaller.

### 3.3. Cognitive and Neuropathological Characteristics between Matched Subtypes

**Figure 5:**
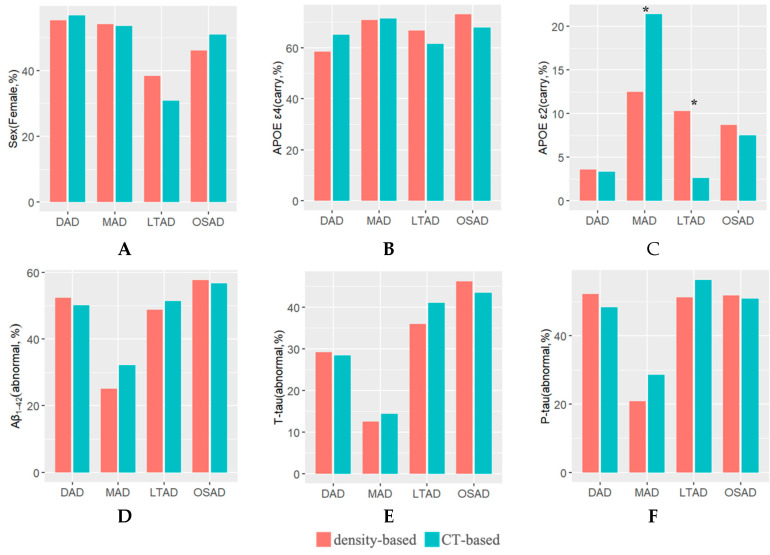
The characteristics of qualitative variables were captured by density features and CT features. The chi-square test was used for comparison between matched subtypes. *: *p* < 0.05 within the matched subtype. (**A**–**C**) The histograms for sex and APOE demonstrate what percentage of AD subjects in each subtype were females and APOE carriers, respectively. (**D**–**F**) The histograms for CSF (Aβ_1-42_, T-tau, and P-tau) indicate the abnormal percentage of AD subjects in each subtype. Abbreviations: AD: Alzheimer’s disease, APOE: apolipoprotein, Aβ_1-42_: beta-amyloid 1-42, P-tau: Phosphorylated tau, T-tau: Total tau. DAD: diffuse atrophy AD subtype, MAD: minimal atrophy AD subtype, LTAD: left temporal dominant atrophy AD subtype, MAD: minimal atrophy AD subtype, OSAD: occipital sparing AD subtype.

**Figure 6:**
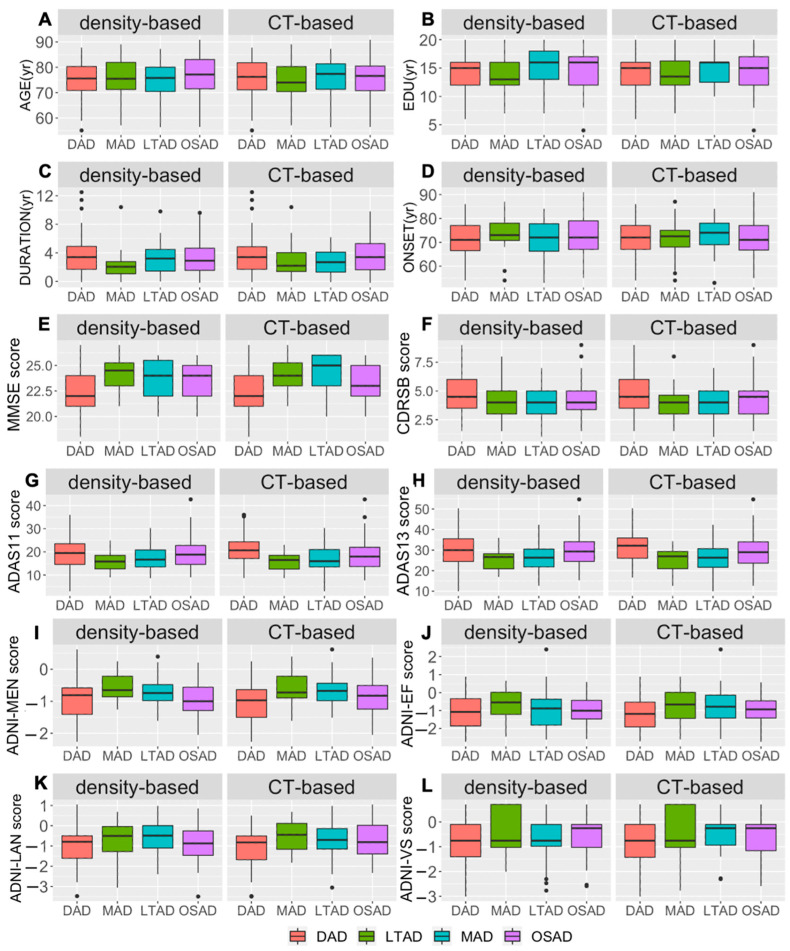
The characteristics of quantitative variables were captured by density features and CT features. Dunnett’s test was conducted comparing the subtypes within each method. (**A**): Age of subjects in each subtype; (**B**): Years of education; (**C**): the duration of AD; (**D**): the onset age of AD, (**E**–**H**): the cognitive scores of MMSE, CDR, ADAS11, and ADAS13; (**I**–**L**): the assessments of ADNI composite score. *Abbreviations:* EDU: education, MMSE: Mini-Mental State Exam, CDRSB: Clinical Dementia Rating Scale-Sum of Boxes, ADAS11: AD Assessment Scale-Cognitive Subscale, which includes 11 tasks, ADAS13: AD Assessment Scale-Cognitive Subscale, which includes 13 tasks, ADNI-MEM: ADNI composite scores for memory, ADNI-EF: ADNI composite scores for executive function, ADNI-LAN: ADNI composite scores for language ability, ADNI-VS: ADNI composite scores for visuospatial ability, yr: year. DAD: diffuse atrophy AD subtype, MAD: minimal atrophy AD subtype, LTAD: left temporal dominant atrophy AD subtype, OSAD: occipital sparing AD subtype.

The characteristics of atrophic regions, demographic, cognitive, APOE genotype (APOE ε2 and APOE ε4), and CSF biomarker levels are described in Figure 5 and Figure 6. And the characteristics of each matched subtype were summarized in Table 2. For each matched subtype, most characteristics of density-based subtypes were consistent with those of CT-based ones. The only difference was reflected in APOE ε2. More APOE ε2 carriers in the MAD_CT than the MAD_density, while more APOE ε2 carriers were observed in LTAD_density than the LTAD_CT. 

## 4. Discussion

This study mainly aimed to test the repeatability and variation of AD subtype definition between density-based and CT-based morphological measures. It transpired that the subtypes generated by both features could morphologically correspond to each other (Figure 3 and Figure 4). There was a good overlap between matched subtypes among these four subtypes in the subjects, and the neuropsychological and pathological characteristics between the matched subtypes were roughly consistent.

In previous studies, neuropathology and neuroimaging have been used to explore AD subtypes. Murray et al. found that the neurofibrillary pathological process in some AD patients followed an alternative distribution based upon a large autopsy series [2]. This was the first time the hypothesis that AD had distinct subtypes from the clinical and pathological points of view had been supported. Whitwell et al. tracked the AD subtypes in vivo by investigating the atrophy patterns in structural MRI, along with regional volumetric analysis [28]. They found three AD subtypes whose atrophy regions matched the neurofibrillary pathological results. Our current study defined the AD subtypes in a univariate way, and the brain regions were treated independently without considering the inter-regional covarying relationship of GM measures. The brain regions with covarying morphological features were observed among the subjects, suggesting shared inter-individual differences in the disease progression [29,30,31]. Atrophy, an established biomarker for neurodegeneration, is related to tau accumulation in certain brain regions. Tau PET imaging provides valuable information regarding tau accumulation in the human brain during aging and neurodegenerative conditions [32]. As indicated in the study, the patterns of hypometabolism noted in each subtype match well with the cortical thinning regions [7]. The aggregation of Aβ_1-42_, a pathological hallmark of AD, has been reported in both cognitively impaired and cognitively normal older adults, although controversies exist over their mechanisms of causing neurodegeneration [33]. The multi-modal neuroimage analysis results revealed that the regional patterns of Aβ_1-42_ deposition, glucose metabolism change, and GM atrophy presented largely overlapping distributions [34,35]. This result accords with the conclusion drawn in a study on AD continuum through the voxel-wise approach, namely, the spatial overlap of pathology and neurodegeneration [36]. The results of our study indicate that the consistency of regional overlap in AD subtype was not markedly influenced by different GM measurement methods.

In the analysis of matched subtype, the only significant difference was presented in the APOE ε2 status of MAD and LTAD. MAD is a special subtype that has attracted widespread attention in previous studies. It has the clinical symptoms of AD, but with no obvious changes in cortical structure compared with normal aging (Figure 3 and Figure 4). In previous studies, mixed results regarding MAD have been presented [37]. Several studies have shown the early onset of MAD [17,38,39]; however, a survey by Byun et al. reported conflicting results [40]. Although some investigators have said that the MMSE score of MAD is the best among all subtypes, an opposite result was obtained in a study [41]. Sometimes it is claimed that the proportion of APOE ε4 carriers is the highest [17,38,39]; however, it was the lowest, as pointed out in various independent studies [41,42]. Abnormal Aβ_1-42_ carriers in MAD accounted for the smallest proportion in Ten Kate’s study [17], but the opposite result has been reported in other studies [38,40]. In our current study, the proportions of APOE ε2 was the most apparent difference in the matched subtype of MAD (Figure 5C). It is widely known that APOE ε4 plays an important role in clearing amyloid-beta peptides, whereas the APOE ε2 variant, found in approximately 5–10% of the population, is considered neuroprotective [43,44]. The young APOE ε2 carriers have higher CT values compared with the other APOE genotypes [45,46]. Elderly normal APOE ε2 carriers also exhibit slower rates of hippocampal atrophy [47]. MAD is a subtype with the lowest degree of atrophy, which may be ascribed to the cortical protective effect of APOE ε2 in the duration of AD. Obviously, the MAD_CT (2.8 ± 2.2 years) had a longer disease duration than MAD density (2.3 ± 2.1 years), and the number of MAD_CT subjects was larger than that MAD density subjects. In addition to the protective effect of APOE ε2, the progression of NFTs cannot be ignored. The spatial distribution of NFTs pathology is associated with neuronal and synaptic dysfunction, and neuronal loss, which initially occur in the transentorhinal cortex and then progress to the hippocampus and other neighboring cortices [1]. Compared with the density features, CT features cannot reflect the measures in subcortical regions. It was inferred that some MAD_CT subjects might not have obvious pathologic changes in cortex, so they were assigned to the MAD subtype.

It was noticed that 79.2% of MAD_CT subjects also belonged to the MAD density, while the rest, 20.8%, were assigned to LTAD density. LTAD presented prominent atrophy in the left lateral parietal, middle, and inferior temporal, with the shortest disease duration. Furthermore, it had the least atrophic degree, except for MAD. Many previous studies have reported that the left hemisphere is more susceptible to AD, whose changes are more severe and may precede the changes in the right hemisphere by up to two years [48,49]. Compared with CT measures, the density morphological measures may be more sensitive to detecting asymmetric subcortical atrophy during the early stage of AD. 

The limitations of this study included: (1) Density features could be obtained through various toolboxes, such as FSL [50], CAT 12 (http://www.neuro.uni-jena.de/cat/, accessed on 3 January 2022), etc. The density features obtained via SPM were used in our study. Whether the processing toolbox could influence the subtype definition was uncertain, which is an interesting issue worthy of further investigation. (2) A further study in a large cohort is required. Here, an ANDI-1 cohort of subjects (around 400 AD or CN subjects) was used to model the definition of subtypes. There were only dozens of patients in each AD subtype. Moreover, due to the absence of nearly half of the data for beta-amyloid and tau, it is difficult to analyze the matched subtypes more deeply from a pathological perspective. (3) Studies on AD continuum show that molecular pathology and neurodegeneration show different spatial relationships in the whole course of AD, so different subtype results may be presented using mode-varying imaging methods in different stages of AD [6,36]. In the future, the influences of different GM features on the definitions of AD subtypes can be further explored. Moreover, the influences of mode-varying imaging methods on the definitions of AD subtypes in different stages of AD can be compared. 

## 5. Conclusions

Two commonly-used neuroimaging morphological measures (CT maps from FreeSurfer’s surface-based calculation and GM density maps from SPM’s voxel-based calculation) in AD subtype definition were used. Next, whether or not the subtypes could be identified consistently with different morphological measures was tested. The results suggest that the two measures can identify relatively consistent subtypes. Because the patterns of atrophy in the matched group appear somewhat consistent, there are likely covarying effects between density and CT measures. For MAD and LTAD, there exists some inconsistency, which may be partially explained through APOE genotype, asymmetric disease progression, and their interactions. However, the definitive resolution will need to await the availability of a larger cohort. The results of our study can be considered as an appropriate reference for facilitating AD subtype definition.

## Figures and Tables

**Table 1:** The parameters selection and evaluation results of MOE.

Method	Optimized Parameters		Evaluation Indicators		
	*t*	*C*	ACC (%)	BPC	W_r_
Density	2	2^−3^	77.3 (4.4)	0.82 (0.03)	0.32 (0.44)
CT	2^−2^	2^−3^	83.1 (4.8)	0.63 (0.02)	0.29 (0.07)

Abbreviations: *t*, *C*: the optimized parameters of MOE, ACC: cross-validated accuracy, BPC: Bezdek partition coefficient, W_r_: maximum pair-wise inner-product; data are presented as mean (standard deviation). CT: cortical thickness.

**Table 2:** A brief description of each subtype.

Subtype	Description	Demographic, Neuropsychological, and Neuropathology Characteristics
DAD	Extensive cortical and subcortical atrophy.	Severe and extensive deficits in all cognitive domains. Higher proportions of APOE ε4 carriers, higher levels of abnormal Aβ_1-42_ and P-tau.
MAD	With the least extent and amount of atrophy in cortical regions, but with sporadic atrophy in subcortical regions.	Good cognitive performance in all fields among the four subtypes. With higher APOE ε2 carriers, and the lowest levels of abnormal Aβ_1-42_, P-tau, and T-tau.
LTAD	Asymmetrical atrophy in the left temporal-parietal cortex.	A relatively low proportion of women, and a higher proportion of APOE ε2 carriers.
OSAD	Prominent atrophy in most of cortex and subcortex, except the occipital area.	The higher levels of abnormal Aβ_1-42_ and T-tau.

Abbreviations: DAD: diffuse atrophy AD subtype, MAD: minimal atrophy AD subtype, LTAD: left temporal dominant atrophy AD subtype, OSAD: occipital sparing AD subtype. APOE: apolipoprotein, Aβ_1-42_: beta-amyloid 1-42, P-tau: Phosphorylated tau, T-tau: Total tau.

## Data Availability

Not applicable.

## Abbreviations

AAL	Automated anatomical atlas	LAN	Language ability
ACC	Cross-validated accuracy	LTAD	left temporal dominant atrophy AD subtype
AD	Alzheimer’s disease	MAD	Minimal atrophy AD subtype
ADAS-Cog	AD assessment scale-cognitive subscale	MEM	Memory ability
ADNI	AD Neuroimaging Initiative	MMSE	Mini-Mental State Examination
ANOVA	Analysis of variance	MOE	Mixture of experts
APOE	Apolipoprotein E	MRI	Magnetic resonance imaging
Aβ_1-42_	Beta-amyloid 1-42	MTL	Medial temporal lobe
BPC	Bezdek partition coefficient	NFTs	Neurofibrillary tangles
CDRSB	Clinical dementia rating scale-sum of boxes	OSAD	Occipital sparing AD subtype
CN	Cognitive normal subjects	P-tau	Phosphorylated tau
CT	Cortical thickness	ROI	Region-of-interest
CSF	Cerebrospinal fluid	SPM	Statistical parametric mapping
DAD	Diffuse atrophy AD subtype	SVM	Support vector machines
EF	Executive function	T-tau	Total tau
FCM	Fuzzy C-Means	VBM	Voxel-based morphometry
GM	Gray matter	VS	Visuospatial ability
ICV	Intracranial volume	W_r_	Maximum pair-wise inner-product
